# Obsessive–Compulsive Disorder with a Religious Focus: An Observational Study

**DOI:** 10.3390/jcm13247575

**Published:** 2024-12-12

**Authors:** Wissam Al Rida Ayoub, Jana Dib El Jalbout, Nancy Maalouf, Samar S. Ayache, Moussa A. Chalah, Ronza Abdel Rassoul

**Affiliations:** 1 Neuroscience Research Center, Faculty of Medical Sciences, Lebanese University, Hadath 1533, Lebanon; wissamalrida.ayoub@lau.edu (W.A.R.A.); ronza.abdelrassoul@gmail.com (R.A.R.); 2 Department of Neurology, Lebanese American University Medical Center-Rizk Hospital, Beirut 1100, Lebanon; nancy.maalouf@lau.edu.lb; 3 Gilbert and Rose-Marie Chagoury School of Medicine, Lebanese American University, Byblos 4504, Lebanon; jana.dibeljalbout@lau.edu (J.D.E.J.); samar.ayache@aphp.fr (S.S.A.); 4 Institut de la Colonne Vertébrale et des NeuroSciences (ICVNS), Centre Médico-Chirurgical Bizet, 75116 Paris, France; 5 EA4391 Excitabilité Nerveuse & Thérapeutique, Université Paris-Est Créteil, 94010 Creteil, France; 6 Department of Clinical Neurophysiology, DMU FIxIT, Henri Mondor University Hospital, Assistance Publique-Hôpitaux de Paris (APHP), 94010 Creteil, France; 7 Pôle Hospitalo-Universitaire Psychiatrie Paris 15, GHU Paris Psychiatrie et Neurosciences, Hôpital Sainte-Anne, 75014 Paris, France

**Keywords:** obsessive–compulsive disorder, religiosity, religious obsessions, spirituality

## Abstract

**Background**: Obsessive–compulsive disorder (OCD) is a psychiatric disorder with poorly detailed subtypes/dimensions, such as religious OCD (ROCD). To date, little is known about ROCD characteristics. This work aimed to describe the sociodemographic and clinical characteristics, along with the religiosity and spirituality, of Lebanese Muslim citizens diagnosed with OCD and exhibiting religious symptoms. **Methods**: Participants were Lebanese Muslims, outpatients with OCD and religious symptoms, aged 18 or above, who could complete a questionnaire. Exclusion criteria were as follows: other psychiatric disorders and cognitive or physical impairments preventing participation. They completed a questionnaire including the 25-item Arabic Scale of Obsessions and Compulsions (10 questions addressing obsessions, 10 questions addressing compulsions, and 5 filler items, all of which were rated on a 4–point Likert scale, with higher total scores indicating increasing severity), the 26-item Spiritual Involvement and Beliefs Scale (rated on a 5-point Likert scale, with higher scores indicating higher spirituality), and questions assessing sociodemographic, clinical, and religiosity variables. **Results**: Fifty adults (62% females, 52% aged between 18 and 29 years) completed the study. They had mild (26%), moderate (48%), and severe (26%) OCD symptoms. The majority attended religious school at least at one point in their life and described a moderate to very high degree of self-religiosity and parental religiosity. Group comparisons (patients with mild vs. moderate vs. severe OCD symptoms) showed significant differences with regard to a family history of psychiatric disorders (*p* = 0.043), the frequency of self-questioning if they prayed correctly (*p* = 0.005), a higher rating of partial ablution repetition (*p* = 0.006), and the frequency of partial ablution repetitions (*p* = 0.041). No significant group differences were noted with regard to sociodemographic or spirituality outcomes. The prevalence of religious doubts (i.e., self-questioning if praying correctly) and specific rituals (partial ablution repetition) among severe OCD patients were 100% (13/13) and 77% (10/13), respectively. **Conclusions**: The results suggest a link between specific religious practices and OCD severity, underscoring the need for culturally sensitive approaches in diagnosing and treating ROCD.

## 1. Introduction

Obsessive–compulsive disorder (OCD) is a heterogeneous psychiatric disorder with a lifetime prevalence of 1–3% [1,2,3]. According to the Diagnostic and Statistical Manual of Mental Disorders (DSM), 5th edition—text revision (DSM-5-TR), OCD belongs to the diagnostic category entitled “Obsessive-compulsive and related disorders” with variable clinical presentations [4,5]. As its name implies, OCD is characterized by the presence of obsessions (e.g., recurrent intrusive unwanted thoughts, images, urges) and/or compulsions (repetitive mental acts or behaviors) aimed to neutralize the obsessions and relieve anxiety. OCD can impose significant burdens on both the social and personal aspects of patients’ lives. Demographically, researchers have found that OCD is more common in females than in males, with an onset of presentation at around 18–29 years of age [5]. While the prevalence of OCD in Lebanese society is not well established, a national epidemiologic survey evaluated the 12-month prevalence of mental disorders in Lebanon and showed a high prevalence of anxiety disorders (11.2%) [6]. In this survey, OCD was considered among the anxiety disorders (according to the 4th edition of the DSM), and its 12-month prevalence was 0.1% [6].

OCD manifests heterogeneously, with several subtypes and symptom dimensions identified. When classifying the subtypes/dimensions based on the focus of compulsions and obsessions, examples could include harm OCD, sexual orientation OCD, pedophilia OCD, relationship OCD, contamination/washing OCD, doubts/checking OCD, symmetry/ordering/arrangement OCD, and scrupulous OCD (or scrupulosity) [7]. The latter subtype is characterized by the presence of religious or moral obsessions and compulsions. When referring to factor-analysis-based studies, a multidimensional model of OCD has been proposed, constituting four or five factors as follows: contamination/cleaning, harmful thoughts, forbidden thoughts (including religious obsessions/rituals), symmetry/ordering/repeating/counting, and hoarding [3].

When evaluating OCD, it is important to take into consideration comorbidities and differential diagnoses [8]. Different OCD dimensions or subtypes might have distinct underlying mechanisms and might respond differently to treatments [2,9]. For instance, religious symptoms are associated with a higher risk of delaying or not starting a treatment, possibly due to important perceived immorality and shame, both of which are related to the heightened response of the amygdala [9].

Neuroimaging studies have linked OCD with pathological findings involving a cortico-basal ganglia-thalamo-cortical loop [10]. OCD management includes psychotherapies (i.e., cognitive behavioral therapies—namely exposure and response prevention), as well as pharmacological treatments (i.e., a selective serotonin reuptake inhibitor (SSRI)) [2] administered in monotherapy or combination. Despite the latter, patients may have a partial or lack of response; such situations are managed by other strategies (e.g., switching the antidepressants to another SSRI/clomipramine, augmentation therapy with antipsychotics, surgical interventions such as deep brain stimulation in severe and pharmaco-resistant cases, or alternative interventions that are gaining growing interest such as noninvasive brain stimulation) [1,2,11,12].

Scrupulosity, defined as “the tendency to be hypervigilant about committing a moral or religious sin” [13], lies at the intersection of OCD symptoms and religiosity. A recent literature review highlights discrepancies and a lack of consensus regarding its definition [14]. Some authors use the terms scrupulosity or scrupulous OCD as overarching categories encompassing (a) secular moral OCD and (b) religious OCD (ROCD), while others use these terms interchangeably [14]. Abramowitz et al. developed a scrupulosity scale that was validated and refined a few years later [15,16]. Several works have assessed scrupulosity in non-clinical and clinical cohorts [17,18,19,20]. For instance, in one work, significant correlations were found between scrupulosity and several outcomes in patients with OCD including obsessive symptoms, an inflated sense of responsibility, beliefs about the importance of and the need to control intrusive thoughts, and moral thought–action fusion (TAF) [17]. In another work involving non-clinical participants, scrupulosity was significantly associated with the use of a specific thought control strategy in response to unwanted intrusions (self-punishment) [18]. Moreover, on the one hand, scrupulosity was significantly associated with several psychopathological symptoms (anxiety, negative affect, disgust sensitivity, specific fears) [16]. On the other hand, scrupulosity appeared to mediate or account for the relationship between obsessive compulsive symptoms and the use of the punishment strategy in non-clinical participants [18,19]. In this current study, the term ROCD will be used.

ROCD involves intrusive blasphemous thoughts, repeating prayers, excessive morality, repetitively asking for reassurance, and unnecessary cleaning habits [20,21,22]. Data on this matter are scarce and sometimes inconsistent. In their research, Himle et al. noted that most of these rituals are performed by the patient to seek forgiveness and atonement for sinful thoughts or actions [21]. While this entity is poorly studied and incompletely elucidated, many studies have investigated potential factors that could be associated with religious obsessions and intrusive thoughts [23,24,25,26,27].

OCD seems to occur with similar frequency across cultures/religions [6,20], although some studies have reported different findings [23]. For instance, across patients with OCD with different religious affiliations, differences were observed in terms of scrupulosity but not OCD symptoms, with higher reporting of scrupulosity in Catholics compared to patients who identified themselves as Protestant, Jewish, or non-religious [20]. Conversely, a greater risk for OCD and higher symptom severity among Catholics were suggested by some authors, while other authors have described more obsessive–compulsive symptom reporting in Muslim patients compared to Christian individuals (for reviews, see [23]).

However, religion might affect the content of symptoms [23]. In other words, OCD symptoms may differ across different religions due to differences in the doctrines and rituals performed [6]. For instance, among devout Muslims, the practice of ablution (washing certain body parts before prayer) may become a compulsive ritual, with individuals repeating the act multiple times to ensure “purity”. A practicing Muslim prays five times a day, performs a cleaning ritual that involves washing certain body parts (i.e., partial ablution) for a predetermined number of times before the prayers, and practices fasting during the holy month of Ramadan [28]. Such religious practices and rituals constitute important issues in Islam and are performed in conformity with strict religious rules [28]. In addition, doubts about religious practices could be perceived as a test of faithfulness (evil temptations), and they are accepted [29]. Moreover, it was found that Catholic individuals are more prone to develop ritualistic ROCD [21], with scrupulosity often manifesting as repeated confessions to seek forgiveness for perceived sins, even minor ones, due to heightened feelings of guilt. Protestants are expected to manifest ROCD with intrusive negative thoughts related to faith [21]. Furthermore, in the Jewish community, OCD manifestation seem to also be shaped by Jewish scriptures [30]. In other words, the pattern of thoughts or actions would be associated with their religious rites and rules.

Besides religion, religiosity might also be associated with the severity of manifestations despite the availability of inconsistent findings [23]. For instance, while no correlation between religiosity and OCD symptom severity was reported by some authors, others have documented more obsessive thoughts and checking, with higher religiosity ratings [23].

There are no clear diagnostic criteria and risk factors listed for ROCD in the literature, and studies conducted thus far have sometimes linked the degree of religiosity to the occurrence of religious obsessions, with no data on the role of sociodemographic variables or past personal/family history of psychiatric illnesses on the development of religious obsessions [31].

Lebanon, a Middle Eastern country, is known for the diverse religious sects encountered within its population, with Islam and Christianity being the two major groups. Moreover, religion is deeply enrooted in Lebanese culture, societal organization, educational system, and political structure [32,33]. As such, one can appreciate the impact that religion has on Lebanese citizens’ decision making in daily life errands and on their personal lives. This relationship might affect citizens with pre-existing OCD by increasing their intrusive thoughts and compulsive behaviors, gearing them toward religious topics, and leaving them with great distress regarding their religious thoughts and rituals. It is also worth mentioning that Lebanon has faced periods of crisis and conflicts, leading to increased stressors and traumatic life events for its population, both of which can predispose individuals to a variety of psychiatric disorders.

This current work addresses religious OCD symptoms, particularly among Lebanese Muslim citizens who were previously diagnosed with OCD. It aims to characterize this clinical population in terms of sociodemographic and clinical characteristics, as well as religiosity and spirituality. It also aims to assess the relationship between the latter features and OCD symptom severity. With the scarcity of data available on this topic and with the Lebanese population being known for its prevalent religiosity and spirituality, making it an adequate target population for studying ROCD, this current study aims to address this gap in the literature and pave the way for future comparative studies to be conducted to establish risk factors and diagnostic criteria for this poorly studied and underreported entity.

## 2. Materials and Methods

This is a prospective cross-sectional pilot study in adult patients with ROCD.

### 2.1. Participants

The participants for this study were recruited through the Lebanese American University Medical Center database. Inclusion criteria were as follows: outpatient adults aged 18 years and above, having a clinical diagnosis of OCD according to the DSM-5-TR diagnostic criteria [5], having religious symptoms, and being able to read and understand the questionnaire.

Exclusion criteria were the presence of other psychiatric disorders or the presence of cognitive or physical impairments preventing participation.

A list of patients with ROCD from the clinical database was prepared. The list was numbered, and the names were masked. Among these patients (n = 217), 50 patients were selected using an online randomizer application (randomizer.org), which generated a dataset of 50 unique numbers.

Afterwards, patients were contacted accordingly, and a visit was planned to check the inclusion/exclusion criteria and provide the patients with adequate information on the study. All participants provided informed consent before engaging in the survey. After accepting to participate, they received by email a link to access the online survey (LimeSurvey, www.limesurvey.org accessed on 9 December 2024).

### 2.2. Survey Instrument

The survey instrument was developed for this research project and was created using an online survey platform, facilitating efficient distribution and data collection.

The survey consisted of multiple sections designed to gather information on participants’ sociodemographic and clinical characteristics, as outlined below: age, sex, relationship status, childhood, and current residence areas, religious vs. nonreligious school, educational level, socioeconomic status, age of diagnosis, pharmacological or psychological interventions, obsessive and compulsive symptoms, and a family history of psychiatric disorders.

The severity of the participants’ OCD was assessed using the Arabic Scale of Obsession Compulsion (ASOC) developed by Abdel-Khalek [34,35,36]. The ASOC was chosen over other scales due to its conception in Arabic. The ASOC is validated in the Arabic language and has good psychometric properties (high internal consistency: α_Cronbach_ = 0.897) [34,36]. Therefore, this scale appears to be relevant for the Lebanese sample.

The ASOC originally consisted of 32 items answered in a yes/no format. The revised version used here consists of 25 items: 10 items addressing obsessions, 10 items addressing compulsions, and 5 filler items to control response bias without being included in the total scores (items #1, #5, #12, #17, and #20). Items are scored on a 4-point Lickert scale (no, some, much, and always). ASOC scores could range from 20 to 80, with 20–39 indicating mild, 40–59 indicating moderate, and 60–80 indicating severe symptoms [34,35,36,37].

Spirituality was assessed using the Spiritual Involvement and Beliefs Scale (SIBS) developed by Hatch and colleagues [38]. The scale involves 26 questions rated on a 5-point scale (strongly agree, agree, neutral, disagree, and strongly disagree) assessing internal beliefs, external practices, personal applications (e.g., practicing humility and forgiveness toward others), and existential and meditative beliefs. Scores vary between 26 and 130, with high scores implying high spirituality [38]. The SIBS is validated in the Arabic language and has good psychometric properties (α_Cronbach_ = 0.76) [39].

The patients’ degree of religiosity was assessed by a questionnaire designed to assess the practice, frequency, and location preference of praying, fasting, partial ablution (washing body parts), and full ablution. The questionnaire also implied questions assessing whether praying/fasting was considered to be performed correctly or accepted by God, and if there are blasphemous thoughts, skeptical thoughts regarding the holy book or prophetic hadiths, or experiencing intrusive thoughts related to ritual impurities (body secretions, blood, flatulence, animal secretions, or shaking hands with a stranger of the opposite sex). The participants were also asked to report on the frequency of visiting religious places and to assess their parents, as well as their own degree of religiosity.

### 2.3. Data Collection Procedure

To collect data, the online survey link was distributed to participants via email through their psychiatrists/psychotherapists. The survey remained accessible for two weeks, allowing participants ample time to complete it. Upon clicking the survey link, participants were directed to a secure online survey platform where they encountered the survey questions. It was a self-administered survey, offering participants the flexibility to complete it at their convenience. To ensure the data’s quality and reliability, participants were required to answer all mandatory questions before submitting the survey. Surveys were anonymous and confidential. It was not possible to track back participants because no identifiers were collected.

### 2.4. Ethical Considerations

Our study adhered to ethical guidelines following IRB approval from the Lebanese American University IRB Office. Participants’ confidentiality and anonymity were strictly maintained throughout the data collection process, collecting no personal identifying information. All collected data were securely stored and accessed only by our research team. As stated above, this study has been reviewed and approved by the LAU IRB (approval date: 17 October 2022) under the following tracking number “LAUMCRH.NM1.17/Oct/2022”.

### 2.5. Data Analysis

Upon completing the data collection phase, survey responses were exported from the online platform into statistical analysis software for further analysis. Data were analyzed using the GraphPad Prism 10.2. First, descriptive statistics were used to present the participants’ sociodemographic, clinical, religiosity, and spirituality data.

Bivariate analysis examined the relationship between the ASOC and sociodemographic and clinical characteristics from the other side. Participants were classified into three groups according to ASOC score categories (mild, moderate, and severe).

The relationship of the ASOC with quantitative data was assessed using the Kruskal–Wallis test (since data did not follow a normal distribution according to the Shapiro–Wilk test) and with qualitative data using Fisher’s exact test.

In addition, a subgroup analysis was performed using the Kruskal–Wallis (in case of three or more categories) or Mann–Whitney test (in case of two categories) to compare the ASOC scores across the different categories of the sociodemographic, clinical, and religiosity variables. For the Kruskal–Wallis test, “***E^2^_R_***” was calculated to provide an estimate of the effect size (***E*^2^*_R_*** *= H/*(N^2^ − 1)(N + 1) where *H* is the test statistic and *N* is the number of observations) [40]. For the Mann–Whitney test, sample size was expressed as “r” which equals Z/√N where Z is the test statistic and N is the number of observations [40]. Due to the exploratory nature of this work, no correction for multiple comparisons was applied.

A correlation analysis was also employed using Spearman’s test to assess the relation between ASOC and SIBS scores.

When group comparisons showed statistically significant differences, regression analyses were conducted. Multiple linear regression was used to examine how independent variables influenced continuous variable ASOC scores. Logistic binary regression assessed the impact of clinical and sociodemographic factors on categorical binary religiosity outcomes.

Quantitative variables were expressed as mean ± SD, and qualitative variables were expressed as number or percentage. *p* values were based on 2-tailed tests, with values <0.05 considered statistically significant.

## 3. Results

### 3.1. Descriptive Statistics

Overall, 50 eligible responses were collected from the online survey. Out of these, 19 (38%) were males, and 31 (62%) were females. Among them, 26 (52%) were aged between 18 and 29 years old, 23 (46%) were between 30 and 49 years old, and only 1 respondent was between 50 and 64 years old. A total of 82% participants lived outside the capital during their childhood, and 80% resided outside the capital at the time of the study. In terms of education, 72% of respondents completed a higher university degree. Regarding socioeconomic status (SES), 46% of the participants earn between 2 and 5 million Lebanese pounds per month, classified as low SES, given the country’s ongoing economic inflation.

Concerning marital status, 48% of them were married, and 46% were never married. Regarding the type of school attended, 52% attended religious schools [exclusively (24%) or at one point in their life (28%)], while 48% reported attending a non-religious school.

Most participants were diagnosed with OCD after the age of 18 years (44% between 18 and 25 years old, 30% after 25 years old) by a mental health specialist. Among them, 54% are not taking any medication and have not considered attending or have attended any psychotherapy sessions. Moreover, a positive family history of psychiatric disorders was common among participants, consisting of OCD (48%), depression (10%), generalized anxiety disorder (6%), or other disorders (4%). A total of 58% reported having experienced traumatic life events.

All participants practiced praying, with the majority doing so daily or weekly (86%). When asked about obsessions related to prayer, 68% reported questioning whether they were praying correctly, and 70% questioned whether their prayers were accepted by God. However, only 44% reported repeating the prayer one (14%), two (22%), or three and more (8%) times to calm obsessive thoughts. Similarly, all participants reported practicing partial ablution, with 50% repeating it one (14%), two (20%), or three times and more (16%). Moreover, most participants (98%) practiced full ablution, with 38% repeating it one (20%), two (6%), or three times and more (12%).

Concerning fasting rituals, 94% reported practicing them during the holy month of Ramadan only (54%), intermittently during the year on special occasions (14%), or 1–6 months per year (26%). A subset of respondents reported questioning whether the fasting was performed correctly (36%) or accepted by God (38%), prompting them to repeat their fast (20%). Additionally, 72% reported experiencing intrusive thoughts related to ritual impurities. A total of 48% reported experiencing them multiple times per day, prompting them to act upon the thoughts sometimes or every time in 68% of the cases.

When asked about blasphemous thoughts or skeptical thoughts regarding holy books, religious scripts, or prophetic sayings, most respondents denied experiencing any.

Participants were asked about their parents, as well as their own degree of religiosity. The majority reported a moderate (46%), high (30%), or very high (8%) degree of religiosity in their parents, as well as a moderate (52%), high (34%), or very high (6%) degree of religiosity in themselves. The majority of the respondents (82%) reported visiting religious places. The ASOC score was 49.82 ± 12.37, and the SIBS score was 81.80 ± 8.85. In this study, the ASOC (α_Cronbach_ = 0.908) and SIBS (α_Cronbach_ = 0.707) had a good internal consistency.

### 3.2. Bivariate Analysis

ASOC scores were compared across the three categories (mild vs. moderate vs. severe OCD symptoms). The Kruskal–Wallis test revealed significant group differences: X^2^ = 41.95, *p* < 0.001. Post hoc Dunn’s test adjusted for multiple comparisons confirmed the significant difference among the three groups: mild vs. moderate OCD symptoms (34.92 ± 2.10 vs. 48.88 ± 5.39, *p* = 0.001), mild vs. severe OCD symptoms (34.92 ± 2.10 vs. 66.46 ± 4.75, *p* < 0.001), and moderate vs. severe OCD symptoms (48.88 ± 5.39 vs. 66.46 ± 4.75, *p* = 0.001).

Data were compared among participants with mild (26%), moderate (48%), and severe symptoms (26%) (i.e., according to the ASOC). No significant differences were observed among the three groups with regard to sociodemographic variables.

**Figure 1:**
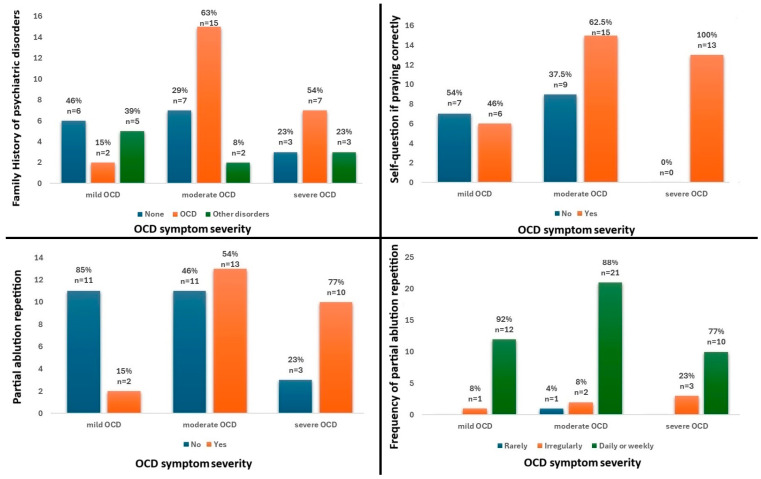
Significant group differences with regard to clinical and religiosity variables among participants with obsessive–compulsive disorder (OCD).

Concerning clinical variables, significant group differences were observed with regard to family history of psychiatric disorders (*p* = 0.043) (Figure 1).

Significant differences were also observed regarding some religiosity variables, namely the frequency of self-questioning if praying correctly (*p* = 0.005), the repetition of partial ablution occurrence (*p* = 0.006), and frequency (*p* = 0.041). Results are summarized in Table 1. Significant findings are presented in Figure 1.

**Figure 2:**
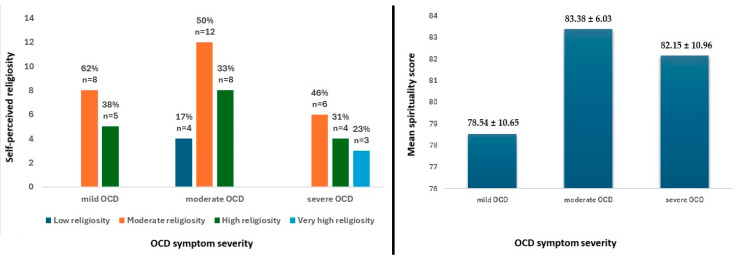
Absence of significant group differences with regard to self-perceived religiosity and spirituality among participants with obsessive–compulsive disorder (OCD).

No significant differences were observed in perceived self-religiosity and spirituality (SIBS) scores among groups (Figure 2).

Subgroup analysis considering ASOC scores as a continuous variable and comparing it among the different categories of each qualitative variable (socio-demographic, clinical, and religiosity variables) yielded significantly higher ASOC scores among patients who questioned themselves if praying correctly compared to those who did not (52.91 ± 12.68 vs. 43.25 ± 8.85, respectively; U = 390.000; *p* = 0.014; effect size r = 0.347) and among patients who repeated partial ablution compared to those who did not (53.68 ± 11.75 vs. 45.96 ± 11.97, respectively; U = 425.500; *p* = 0.028; effect size r= 0.310). No other significant results were observed. Data are summarized in Table 2.

Stepwise linear regression was conducted to predict ASOC scores (dependent variable) using the following independent variables: family history of psychiatric disorders, self-questioning about praying correctly, and partial ablution repetition. The results showed that only self-questioning about praying correctly significantly contributed to the model (F(1,48) = 7.519, *p* = 0.009), explaining 13.5% of the variance in ASOC scores (standardized β = 0.368).

Logistic binary regression was used to analyze the effects of clinical and sociodemographic variables on religiosity, focusing on two dependent variables: (a) self-questioning about correct prayer and (b) partial ablution repetition. Only the ASOC category significantly increased the likelihood of self-questioning prayer correctness (OR = 4.21, 95% CI [1.62; 13.21]). The model fit well, with a significant log-likelihood ratio test (9.31, *p* = 0.002) and a pseudo-R^2^ value (Nagelkerke R^2^ = 0.24), explaining 24% of the variability in this religiosity outcome. Similarly, only the ASOC category increased the likelihood of repeating partial ablutions (OR = 4.20, 95% CI [1.71; 12.58]). This model also showed good fit (test statistic: 10.59, *p* = 0.001) and explained 25% of the variability in the dependent variable.

Finally, no significant correlation was found between ASOC and SIBS scores (ρ = 0.121, *p* = 0.402).

## 4. Discussion

In this study, we described the sociodemographic characteristics, clinical variables, religiosity, and spirituality among patients diagnosed with OCD exhibiting ROCD symptoms, and we assessed the relationship between these variables and OCD symptom severity.

### 4.1. Sociodemographic and Clinical Characteristics

This study included 50 participants, with a higher representation of females than males, consistent with findings from a previous study performed in Morocco [31]. Most of them were aged between 18 and 29 years, followed by the 30–49-year-age group, and a smaller proportion from the 50–64-year-age group. They were mostly diagnosed after 18 years of age. These findings align with the literature where, despite mixed findings, OCD seems to be more frequent among adult females while it seems to be more common in male children [41]. Females seem to have an older age of onset compared to males [42]. Group comparison showed no significant sex differences according to OCD symptom severity, in line with some previous findings on this matter [42]. Conversely, in terms of ROCD, some studies suggest ROCD symptoms to be more frequent in males compared to females [43].

Most of the cohort attended religious schools at least at one point in their life and described a certain degree of parents’ religiosity. Limited data are available regarding the relationship between OCD manifestation and cultural or educational background [23]. With some exceptions, the available data suggest a higher frequency of religious themes in Middle Eastern countries compared to Occidental and Far-East countries [30]. Culture may not only shape OCD symptoms (e.g., the content of obsessions and the expression of compulsions) but also influence patients’ interpretations of their symptoms and their approach to symptom management [30,44]. These merits further assessment.

Significant group differences were observed regarding the family history of psychiatric disorders, and 68% had a positive family history of psychiatric disorders (most frequently OCD). However, statistical significance was not reached in subgroup analysis, a finding that could be attributed to the small sample size. A high risk of OCD in first-degree relatives was previously reported [45,46]. For instance, a risk of ~23% (odds ratios: 11–32) was reported in controlled family studies [45]. Moreover, in a nationwide study including 23,258,175 participants with OCD and 89,500 first-degree relatives, a high relative risk of OCD was observed among first-degree relatives (relative risk: 8.11) [46].

More than half of the participants reported having experienced traumatic life events. Some evidence suggests a relationship between traumatic life events and OCD [47,48,49]. In previous works, traumatic life events were reported by 54–60.8% of patients with OCD [47,48]. Some authors suggest that experiencing traumatic incidents is common in late-onset OCD compared to early-onset genetic OCD [49]. In addition, in a large meta-analysis involving 24 articles (4557 participants), a significant association was observed between traumatic life events and OCD symptom severity [50]. Interestingly, this relationship was found to be stronger in females than in males, and the association concerns the severity of compulsions but not obsessions [50].

### 4.2. Religiosity and Spirituality

In this work, most participants reported a certain degree of religiosity (moderate to very high). Additionally, a significant proportion reported regular visits to religious places. Religious practices were also prevalent, with almost all participants reporting engagement in daily or weekly prayer (86%), practicing ablution (98%), and fasting rituals (94%) during Ramadan or other occasions. This demonstrates the importance of religious practices in their lives.

Obsessions related to religious practices were also assessed. Many participants reported frequent doubts about whether they were praying correctly (68%) and whether their prayers were accepted by God (70%). A subset of patients (44%) reported engaging in repetitive prayer to alleviate obsessive thoughts. Regarding ritual impurities, most participants (72%) reported experiencing intrusive thoughts related to specific ritual impurities, leading them to perform compulsive actions.

When comparing patients according to their OCD symptom severity, significant group differences were observed regarding the frequency of self-questioning if praying correctly, the occurrence of partial ablution repetition, and the frequency of this practice. In addition, the subgroup analysis revealed significantly higher ASOC scores among patients who questioned themselves if praying correctly and repeated partial ablution compared to those who did not. ASOC categories significantly predicted both religious outcomes. Additionally, self-questioning if praying correctly was the only significant predictor of ASOC scores.

Such results are consistent with those of previous studies. In one study from Morocco, self-questioning if praying correctly was the most common religious obsession affecting 47% of patients [31]. In another work from Saudi Arabia, half of the obsessions concerned prayers and the associated washing rituals [51].

In fact, no causality link seems to exist between religiosity/spirituality and OCD occurrence. However, in religious individuals, the symptoms would manifest with religious themes [52]. Particularly, the symptoms’ expression may be related to the religion in question, such as self-questioning if praying correctly or repeating washing rituals in the case of Muslims.

In addition, as found in this present work, a relationship seems to exist between the degree of religiosity/spirituality and the intensity of symptoms [29,44,53]. For instance, in a group of Muslim and Christian subjects from Turkey and Canada, Yorulmaz and colleagues found an association between religiosity ratings (based on the Religiousness Screening Questionnaire) and the frequency of obsessive thoughts and checking (evaluated by means of the Obsessive–Compulsive Beliefs Questionnaire) [29]. In another work by Rakesh and colleagues involving patients with OCD from India, a significant correlation was found between religiosity (Belief into Action Scale) and OCD symptoms severity (Yale-Brown Obsessive–Compulsive Scale) [53].

In some works, this relationship between religiosity and OCD symptom severity seems to be moderated by some variables such as disgust sensitivity or thought–action fusion [54,55]. The latter consists of maladaptive cognitions about the link between mental events and behaviors (believing that thoughts are morally equivalent to action), which could be associated with the teaching that may underlie some religious doctrines [54,55]. Thought–action fusion might induce thought suppression, which could subsequently promote symptoms [56].

### 4.3. Limitations and Implications

It is important to consider the limitations of this study. As it is a pilot study, the sample size was relatively small and not representative of the entire Lebanese population, which could limit the generalizability of the study results. This study relied on self-reported measures, which can be subject to recall bias. Moreover, the choice of an online survey can introduce response biases, especially in studies about sensitive topics like religious beliefs. Some respondents may under-report or over-report symptoms due to social desirability. Additionally, this study did not include a control group. The lack of a control group limits the ability to determine if the observed behaviors are significantly higher than in the general population. Including a control group in future studies could reveal if the relationship between religious practices and OCD symptoms is unique to ROCD patients or present to a lesser degree in religious individuals without OCD. Moreover, the study focused on one religious group (Muslims), limiting the applicability of the results to other religions. Furthermore, this study assessed the current pharmacological status of patients, as well as the current or past management with psychotherapies, but it did not collect the therapeutics of the prior months (treatment type, dosage, initiation date) in detail. Although there are no group differences with regard to the treatment status (subgroup analysis results), the existence of prior therapeutic interventions could mitigate the severity and manifestation of key ROCD symptoms and might modify the results. However, data were presented without corrections for multiple comparisons, potentially increasing the risk of Type I errors (false positives).

Despite these limitations, this study provides valuable preliminary findings that shed light on the demographic characteristics, mental health symptoms, religious practices, and religious obsessions among a specific group of Lebanese individuals. These findings may contribute to the understanding of the intersection between religiosity and mental health and inform future research and interventions in this domain. Further studies with larger and more diverse samples are necessary to validate and expand upon these initial findings.

Future investigations into ROCD could benefit from considering some clinical, cognitive and affective factors that might influence symptom expression and treatment outcomes [57,58,59,60,61,62,63,64]. For instance, some affective temperaments (cyclothymic, anxious, depressive) could play a role in emotional instability, OCD presentation, and treatment response, warranting deeper exploration within the specific framework of ROCD [57]. Moreover, in some studies, patients with ROCD manifested significantly higher obsessive–compulsive personality traits or disorder, as well as depressive symptoms or psychotic processes, compared to patients with other OCD subtypes or healthy controls [25,26,27].

Finally, it is worth noting that the findings of this work could have clinical implications. It would be beneficial to include an assessment of religious practices as part of the OCD diagnostic process in religious contexts, examining how certain behaviors (e.g., ablution or prayer frequency) may distinguish between religious observance and compulsive repetition. Culturally sensitive interventions appear pertinent, including collaboration with religious figures or community leaders when needed to help patients understand their symptoms from a medical and religious perspectives and harmonize mental health care with faith-based values [65]. Adapting evidence-based therapies, such as exposure and response prevention, to align with patients’ religious beliefs could enhance treatment efficacy and improve patient adherence [65].

## 5. Conclusions

The main finding of this work is a significant association between OCD symptoms severity and some religiosity variables, namely self-questioning if praying correctly and partial ablution repetition. This study provides insights into the characteristics of patients exhibiting ROCD. The findings show that religious practices are significant in their lives, and religious obsessions are not uncommon. Although this study has limitations, it opens the door for further research in this under-explored area of mental health. Mental health professionals should be aware of religious obsessions as a potential aspect of OCD symptomatology, approaching it with cultural sensitivity. It appears pertinent to assess religious practices as part of the OCD diagnostic process in religious contexts, particularly focusing on how ritualistic behaviors like ablution or prayer frequency may vary between religious observance and compulsive repetition. A deeper understanding of the interplay between religiosity and OCD may improve mental health care strategy and provide culturally sensitive patient care.

## Figures and Tables

**Table 1:** Comparison of sociodemographic, clinical, religiosity, and spirituality variables among patients with mild, moderate, and severe obsessive–compulsive symptoms. Data are presented in terms of the number of participants, except for SIBS scores, which are presented as mean ± SD. USD: United States dollar. Bolded p values are statistically significant (*p* < 0.05).

	Mild (n = 13)	Moderate (n = 24)	Severe (n = 13)	*p* Value
Age				0.489
18–29 years	5	13	8	
30–49 years	7	11	5	
≥50 years	1	0	0	
Sex				0.162
Females	5	17	9	
Males	8	7	4	
Educational level				0.057
Middle school	1	1	1	
High school	2	9	0	
University degree	10	14	12	
Relationship status				0.713
Single	6	13	4	
Married	6	10	8	
Divorced	1	1	1	
Area of living (childhood)				0.897
Outside the capital	10	20	11	
Within the capital	3	4	2	
Area of living (current)				>0.999
Outside the capital	10	19	11	
Within the capital	3	5	2	
Monthly living income (Lebanese pounds and equal rates in USD) <2 millions (~22 USD) Between 2 and 5 millions (~22–56 USD) Between 5 and 10 millions (56–112 USD) Between 10 and 20 millions (112–224 USD) >20 millions (>224 USD)	0 3 4 2 4	3 14 2 3 2	2 6 1 2 2	0.240
Attended school				0.913
Religious	3	5	4	
Non-religious	6	13	5	
Both	4	6	4	
Parents’ relationship status				0.65
Married	12	18	11	
Divorced	0	4	1	
Widowed	1	2	1	
Traumatic life events				0.662
No	7	9	5	
Yes	6	15	8	
Age of diagnosis				0.184
Before 12 years	2	1	1	
12–18 years	0	8	1	
18–25 years	6	9	7	
>25 years	5	6	4	
Family history of psychiatric illness				**0.043**
None	6	7	3	
OCD	2	15	7	
Others	5	2	3	
Current OCD medications				0.119
Untreated	7	10	10	
Treated	6	14	3	
Current or past psychotherapy				0.482
None	9	12	6	
Past	1	7	5	
Current	3	5	2	
Praying frequency				0.702
Rarely	0	1	0	
Irregularly	1	2	3	
Daily or weekly	12	21	10	
Self-question if praying correctly				**0.005**
No	7	9	0	
Yes	6	15	13	
Self-question if prayers are accepted by God				0.111
No	4	10	1	
Yes	9	14	12	
Frequency of prayers repetition if questioning				0.162
None	9	14	5	
Once	1	2	5	
Twice	1	6	3	
Three of more	2	2	0	
Praying location				0.830
No preference	7	16	6	
As long as the setting is available	1	1	2	
Home only	3	4	2	
Home and praying place	2	3	3	
Frequency of partial ablution				0.404
Daily 3–5/day	12	18	12	
More than 5/day	1	6	1	
Partial ablution repetition				**0.006**
No	11	11	3	
Yes	2	13	10	
Frequency of partial ablution repetition				**0.041**
None	11	11	3	
Once	1	2	4	
Twice	1	6	3	
Three or more	0	5	3	
Partial ablution location				0.058
No preference	6	15	4	
Home only	5	9	5	
Home and praying place	2	0	4	
Full ablution repetition				0.123
No	10	16	5	
Yes	3	8	8	
Frequency of full ablution repetition				0.166
None	10	16	5	
Once	2	4	4	
Twice	1	2	0	
Three of more	0	2	4	
Frequency of fasting practice				0.252
None	0	3	0	
During the holy month	10	11	6	
1–6 months per year	3	5	5	
Throughout the year during religious ceremony	0	5	2	
Self-question if fasting correctly				0.282
No	10	16	6	
Yes	3	8	7	
Self-question if fasting accepted by God				0.703
No	9	15	7	
Yes	4	9	6	
Frequency of fasting repetition				0.495
None	10	21	9	
Rarely	2	1	1	
Sometimes	1	2	3	
Frequency of suspecting intrusive thoughts related to ritual impurity				0.365
None	6	6	2	
Sometimes	1	3	1	
>once per month	0	1	0	
>once per week	3	2	1	
>once per day	3	12	9	
Frequency of attempts to correct suspected ritual impurities				0.445
None	6	6	2	
Rarely	1	1	0	
Sometimes	1	4	1	
Every time	5	13	10	
Blasphemous thoughts				0.459
No	10	13	8	
Yes	3	11	5	
Skeptical thoughts regarding the holy book				>0.999
No	11	19	11	
Yes	2	5	2	
Skeptical thoughts regarding religious scripts or prophetic Hadiths				>0.999
No	8	16	9	
Yes	5	8	4	
Perceived parents’ religiosity				0.221
No Practice	0	0	1	
Low	2	5	0	
moderate	7	11	5	
High	3	5	7	
Very high	1	3	0	
Perceived self-religiosity				0.104
Low	0	4	0	
moderate	8	12	6	
High	5	8	4	
Very high	0	0	3	
Frequency of visiting religious places/centers				0.864
None	2	6	1	
Rarely	2	1	1	
Religious occasions	3	3	4	
Sometimes	2	6	2	
Weekly	3	7	4	
Daily	1	1	1	
SIBS scores	78.54 ± 10.65	83.38 ± 6.03	82.15 ± 10.96	0.277

**Table 2:** Subgroup analysis comparing the obsessive–compulsive symptoms score (ASOC) among the different categories of the sociodemographic, clinical, and religiosity variables. Bolded *p* values are statistically significant (<0.05).

	Mann–Whitney or Kruskal–Wallis Test Statistics	*p* Value	Effect Size (r or E^2^_R_)
Age	*H* = 3.044	0.218	0.062
Sex	*U* = 232.000	0.211	0.177
Educational level	*H* = 2.845	0.416	0.008
Relationship status	*H* = 0.248	0.884	0.005
Area of living (childhood)	*U* = 180.500	0.921	0.014
Area of living (current)	*U* = 193.000	0.877	0.024
Monthly living income (Lebanese pounds)	*H* = 4.266	0.371	0.087
Attended school	*H* = 0.915	0.633	0.019
Parents’ relationship status	*H* = 0.120	0.942	0.002
Traumatic life events	*U* = 365.500	0.230	0.170
Age of diagnosis	*H* = 0.611	0.894	0.012
Family history of psychiatric illness	*H* = 4.419	0.110	0.090
Current OCD medications	*U* = 248.00	0.223	0.172
Current or past psychotherapy	*H* = 1.665	0.435	0.034
Praying frequency	*H* = 1.386	0.500	0.028
Self-question if praying correctly	*U* = 390.000	**0.014**	0.347
Self-question if prayers are accepted by God	*U* = 300.000	0.427	0.112
Frequency of prayers repetition if questioning	*H* = 3.552	0.314	0.072
Praying location	*H* = 1.506	0.681	0.031
Frequency of partial ablution	*U* = 189.000	0.594	0.079
Partial ablution repetition	*U* = 425.500	**0.028**	0.310
Frequency of partial ablution repetition	*H* = 6.785	0.079	0.138
Partial ablution location	*H* = 0.600	0.741	0.012
Full ablution repetition	*U* = 13.000	0.560	0.113
Frequency of full ablution repetition	*H* = 6.832	0.077	0.139
Frequency of fasting practice	*H* = 1.120	0.772	0.023
Self-question if fasting correctly	*U* = 368.500	0.103	0.230
Self-question if fasting accepted by God	*U* = 342.000	0.342	0.134
Frequency of fasting repetition	*H* = 1.038	0.595	0.021
Frequency of suspecting intrusive thoughts related to ritual impurity	*H* = 3.653	0.455	0.075
Frequency of attempts to correct suspected ritual impurities	*H* = 1.933	0.586	0.039
Blasphemous thoughts	*U* = 338.500	0.379	0.124
Skeptical thoughts regarding the holy book	*U* = 209.500	0.534	0.089
Skeptical thoughts regarding religious scripts or prophetic hadiths	*U* = 289.500	0.854	0.026
Perceived parents’ religiosity	*H* = 7.912	0.095	0.161
Perceived self-religiosity	*H* = 3.146	0.370	0.064
Frequency of visiting religious places/centers	*H* = 1.533	0.199	0.031

## Data Availability

Data can be made available by the corresponding author upon reasonable requests.
